# Identification of a novel PANoptosis-related gene signature for predicting the prognosis in clear cell renal cell carcinoma

**DOI:** 10.1097/MD.0000000000039874

**Published:** 2024-09-27

**Authors:** Dezhi Yue, Congzhe Ren, Hu Li, Xiaoqiang Liu

**Affiliations:** a Department of Urology, Tianjin Medical University General Hospital, Tianjin, China; b Department of Urology, Shanxian Central Hospital, Heze, Shandong, China.

**Keywords:** clear cell renal cell carcinoma, nomogram, PANoptosis, risk score, signature

## Abstract

PANoptosis has been shown to play an important role in tumorigenesis and gain more attention. Yet, the prognostic significance of PANoptosis-related genes has not been investigated more in clear cell renal cell carcinoma (ccRCC). The aim of this research was designed to identify and create a signature of PANoptosis-related genes which was expected to predict prognosis of ccRCC more effectively. The transcriptome data and clinical information were collected from The Cancer Genome Atlas database and the Gene Expression Omnibus database. Optimal differentially expressed PANoptosis-related genes, which were closely associated with prognosis and employed to construct a risk score, were extracted by univariate Cox analysis, least absolute shrinkage and selection operator Cox regression and multivariate Cox analysis. We performed Kaplan–Meier survival analysis and time-dependent receiver operating characteristic curves to complete this process. By adopting univariate and multivariate analysis, the constructed risk score was assessed to verify whether it could be taken as an independent contributor for prognosis. Moreover, we created a nomogram in order to predict overall survival (OS) of ccRCC. Five differentially expressed PANoptosis-related genes were screened out and used to construct a risk score. Our results showed that ccRCC patients with high risk score had a poor prognosis and shorter OS. The results of Kaplan–Meier curves and the area under the receiver operating characteristic curves of 1-, 3-, and 5-year OS indicated that the prediction performance was satisfactory. Additionally, the risk model could be taken as an independent prognostic factor in training and validation cohorts. The nomogram exhibited excellent reliability in predicting OS, which was validated by calibration curves. We identified 5 PANoptosis-related genes, which were used to construct a risk score and a nomogram for prognostic prediction with reliable predictive capability. The present study may provide new potential therapeutic targets and precise treatment strategies for ccRCC.

## 1. Introduction

Renal cell carcinoma (RCC), which is considered to be one of the most prevalent tumors in urinary system, exhibits highly malignant and lethal characteristics.^[1]^ According to the statistics in 2020, more than 430,000 RCC cases were newly diagnosed and nearly 200,000 deaths occurred worldwidely.^[2]^ This indicated RCC has become a serious threat globally. With asymptomatic feature in the early stages, ¼ to ⅓ of RCC patients have missed the optimal opportunity for treatment because of distant metastases having occurred when diagnosed.^[3]^ As the most common histopathological subtype, clear cell renal cell carcinoma (ccRCC) is responsible for up to 75% in RCC patients.^[4]^ It has been confirmed that ccRCC is of high tumor heterogeneity and rather aggressive.^[5]^ With great advances in treatment methods, alternative therapies, such as surgery, immunotherapy and targeted therapy, have made major breakthroughs.^[6]^ However, the prognosis and progression-free survival of patients in late stages remain unsatisfied. Consequently, considering the poor prognosis and limitation of the ccRCC patients, it is critical to construct a more credible and adaptable predictive model to evaluate the prognosis effectively and facilitate optimal therapeutic alternatives.

Identified as a novel form of programmed cell death (PCD), PANoptosis, comprises the major features and characteristics of pyroptosis, apoptosis, and necroptosis.^[7]^ This uncommon PCD has attracted increasing attention recent years and enriched the understandings of PCD. PCD are types of regulated cell death pathways mediated by upstream receptors and signaling cascades, which contribute to the resistance to cellular insults and homeostasis maintenance for the host.^[8]^ When the organism senses an endogenous and exogenous stimulation, PCD pathways are activated and executed to eliminate lesions to the host.^[9]^ Up to date, pyroptosis, apoptosis and necroptosis are the most intensively studied models of cell death pathways.^[10,11]^ Previous studies have considered these 3 pathways as mutually dependent and parallel models of cell death with no overlap, functioning in separate mechanisms. However, increasing evidence confirms that there is much crosstalk and coordination among pyroptosis, apoptosis and necroptosis pathways.^[12,13]^ For example, activated caspase-8 (CASP8), which is recognized as the initiator and promoter of apoptosis, could restrain necroptosis pathway by inhibiting RIPK1.^[14]^ NOD-like receptor family pyrin domain containing 3 (NLRP3) inflammasomes, contributing importantly to pyroptosis pathway, can promote the production and release of the key pyroptotic cytokines.^[15]^ Additionally, CASP8 can mediate the cleavage of gasdermin D (GSDMD), assembly of NLRP3 inflammasomes as well as production of IL-1β, indicating that CASP8 also can perform a critical role in pyroptosis pathway.^[16]^ The above studies suggest that these 3 cell death pathways mutually interact and coordinate with each other, forming a complex network of molecular mechanisms.

Numerous advanced researches suggest that PANoptosis exerts a crucial role in the initiation and progression in a wide range of diseases, such as infectious disorders, neurodegenerative lesions, ischemia–reperfusion injuries and inflammatory diseases.^[17–20]^ Simultaneously, increasing studies have confirmed the significance of PANoptosis regulation in a number of cancers, for instance, colorectal cancer (CRC), adrenocortical cancer, low-grade gliomas and gastric cancer.^[21–24]^ However, PANoptosis-related studies are inadequate in ccRCC patients. Since the increasingly critical role of PANoptosis, further researches in ccRCC may provide a novel insight for therapeutic approaches and prediction of prognosis. By a comprehensive data analysis from The Cancer Genome Atlas (TCGA) database, our study built a PANoptosis-related gene signature that demonstrated a quite satisfactory prognostic prediction value for ccRCC patients. And we further performed validation in the Gene Expression Omnibus (GEO) database, in order to confirm whether this PANoptosis-related gene signature could exhibit the same predictive capability in other databases as well.

## 2. Materials and methods

### 2.1. Data collection and processing

All the gene transcriptome profiles and the corresponding clinical characteristics of ccRCC were obtained from the TCGA database (https://portal.gdc.cancer.gov/). Duplicated data, samples with overall survival (OS) <30 days and missing clinical information were excluded. The mRNA transcriptome data of protein coding genes were retained. Then, we got a cohort of 511 ccRCC patients with the mRNA transcriptome and clinical information, as the training cohort, for the following analysis. We downloaded the ESTIMATE, stromal and immune scores corresponding to the ccRCC patients of the TCGA from the ESTIMATE database (https://bioinformatics.mdanderson.org/estimate/index.html). Based on the immune score of 0 as the cutoff threshold, all ccRCC patients were classified into low and high immune score groups. The dataset GSE167573 including completed transcriptome data and clinical information was obtained from the GEO database (http://www.ncbi.nlm.nih.gov/geo/).

The data analysis was executed by using the “*edge*” R package through the R software in the training cohort. We took false discovery rate (FDR) < .05 as well as |log2fold change (FC)| > 2 as selection criteria. Then differentially expressed genes (DEGs) were screened between the 2 groups stratified by immune score. In order to exhibit the DEGs, we plotted a volcano map by “*ggplot2*” package. The PANoptosis-related genes (PANRGs) list was collected from the previous researches. Additionally, Venn diagram was preliminarily employed to screen the potential prognostic differentially expressed PANoptosis-related genes (DEPANRGs).

### 2.2. Functional enrichment analysis

For exploring the most relevant function of the preliminarily screened DEPANRGs, we adopted the Database for Annotation, Visualization and Integrated Discovery (DAVID) database (https://david.ncifcrf.gov/) to complete functional annotation in our study. We uploaded the DEPANRGs list and implemented Gene Ontology (GO) analysis. In parallel, we completed Kyoto Encyclopedia of Genes Genomes (KEGG) analysis to annotate relevant pathways for the DEPANRGs.

### 2.3. Correlation analysis

In order to further investigate the interaction among the proteins encoded by the DEPANRGs, we performed protein–protein interaction (PPI) analysis and downloaded matched data of these DEPANRGs from the STRING database (version 11.5, https://cn.string-db.org/). Subsequently, the Cytoscape software (version 3.9.1) was employed to create a PPI network to reveal the interaction of them. The “*ggcorrplot*” and “*corrplot*” packages were used to perform Spearman analysis and visualize the correlation of the DEPANRGs.

### 2.4. Construction and verification of a DEPANRGs risk score

To explore the prognosis-related genes, the univariate Cox analysis was performed by using survival package and then 13 genes of the DEPANRGs with *P* value < .05 were screened out. The optimal DEPANRGs, which were closely relevant to the prognosis of ccRCC patients, were identified by employing the least absolute shrinkage and selection operator (LASSO) Cox regression via application of “*glmnet*” package to obtain the optimal *λ* value. Subsequently, after executing the multivariate Cox analysis by “*survival*” package, the most key DEPANRGs were screened out with obtainment of regression coefficients. Then the coefficients were employed to generate a risk score, as shown below:


$$\begin{array}{l} Risk\;\;\;score=\Sigma i\left(\beta i\times DEPANRGi\right). \end{array}$$

The *β* means the coefficient of the DEPANRGs, while DEPANRG means the expression levels of these genes. Based on median value of the generated risk score as stratification criteria, ccRCC patients of the training and validation cohorts were classified into low risk and high risk groups, respectively. The principal component analysis was generated by using “*scatterplot3d*” package to verify the discriminating ability. By using “*survminer*” package, Kaplan–Meier (K–M) survival analysis and a long-rank test was performed to compare the survival rate between groups stratified by risk score. With employment of “*timeROC*” and “*survival*” packages, Time-dependent receiver operating characteristic (ROC) curves were taken to estimate the predictive capability.

### 2.5. Construction and calibration of a nomogram

To further evaluate predictive value of the risk score and clinical characteristics, we performed univariate and multivariate Cox analysis to verify whether the risk score could be used as an independent predictive factor. Moreover, in order to predict survival rates for ccRCC patients, the “*rms*” package was employed for construction of a nomogram combining the risk score and clinical characteristic data from the TCGA. For determining the prediction consistency of the constructed nomogram, calibration curves were obtained by using the “*rms*” package.

### 2.6. Immune infiltration analysis

The ESTIMATE, stromal and immune scores matched the patients from the TCGA database were downloaded for following immune analysis. Additionally, we investigated the distribution of immune checkpoints expression in groups stratified by risk score. TIMER database, which is recognized as a reliable database for analyzing the abundance of immune infiltration, was utilized to study the correlation between DEPANRGs and immune infiltration cells.

### 2.7. Statistical analysis

Using a series of packages, statistical analysis was conducted by the R software (version 4.2.2). We chose Student *t* test to statistically analyze DEPANRGs expression. *P* value < .05, which was considered to be cutoff value, was employed to determine significant difference.

## 3. Results

### 3.1. Identification of DEPANRGs

**Figure 1.**
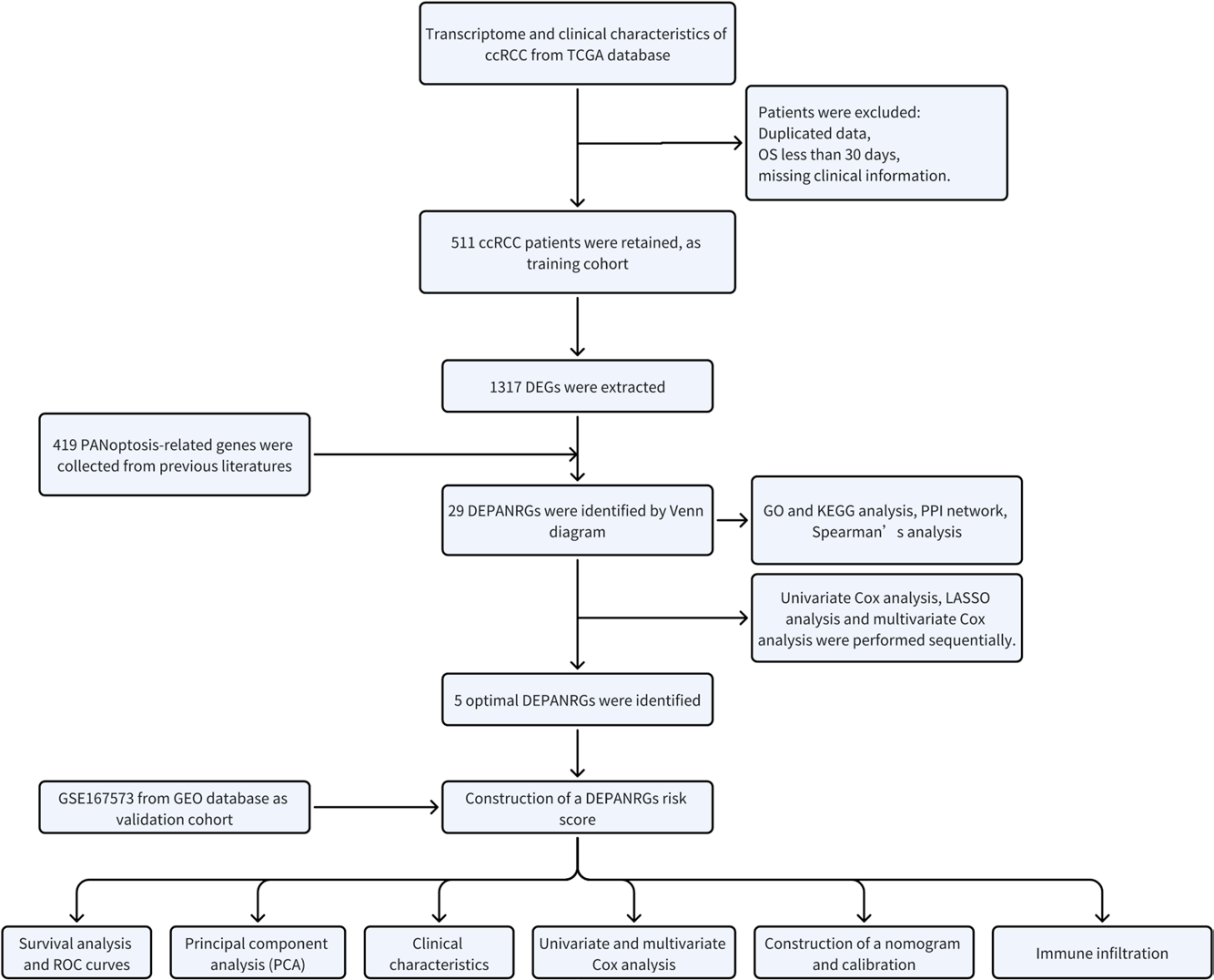
The flow diagram of this study.

**Figure 2.**
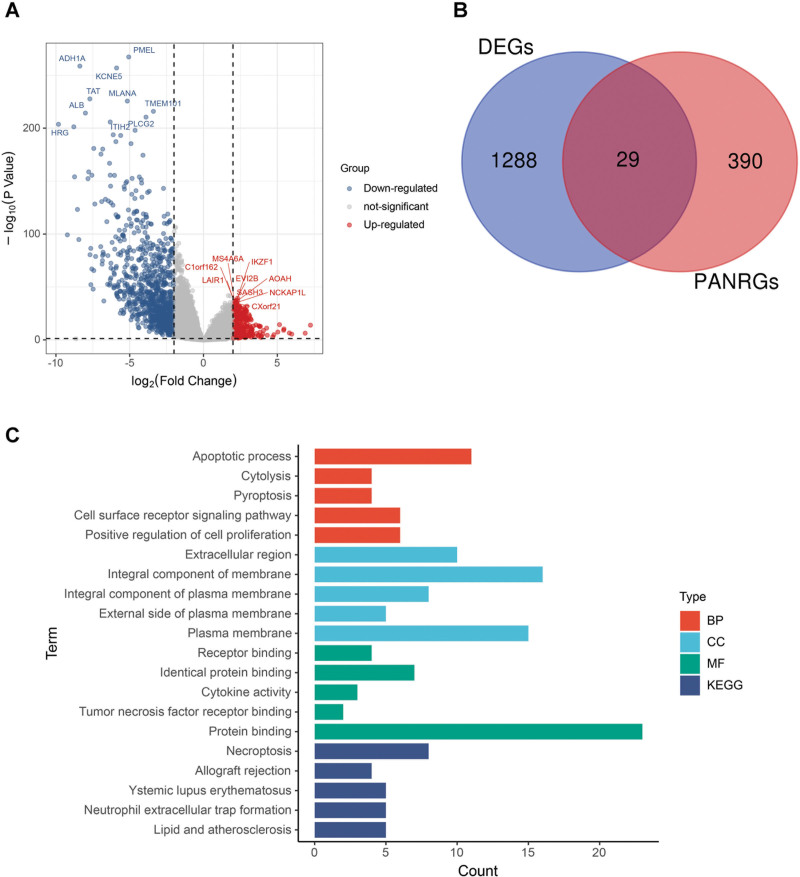
Identification of DEGs. (A) The volcano plot of 1317 DEGs between the low and high immune score groups of ccRCC from the TCGA. (B) The Venn diagram showing 29 DEPANRGs. (C) GO analysis and KEGG pathway analysis of the 29 DEPANRGs. ccRCC = clear cell renal cell carcinoma, DEG = differentially expressed gene, DEPANRG = differentially expressed PANoptosis-related gene, GO = Gene Ontology, KEGG = Kyoto Encyclopedia of Genes Genomes, TCGA = The Cancer Genome Atlas.

The flow diagram was showed in Figure 1. Taking FDR < .05 and |log2FC| > 2 as the selection criteria, we extracted 1317 differentially expressed genes (DEGs) between groups stratified by immune score, including 309 upregulated genes and 1008 down-regulated genes (Fig. 2A). A total of 419 PANoptosis-related genes were collected from previous literatures. Through Venn diagram, 29 differentially expressed PANRGs (DEPANRGs) were identified and represented in Figure 2B.

### 3.2. Functional enrichment analysis of DEPANRGs

For exploration and determination of the DEPANRGs-associated biological function, Gene Ontology analysis and KEGG pathways of the 29 DEPANRGs were implemented in the DAVID database. The top 5 terms were visualized in Figure 2C. The top 5 enriched biologic processes were apoptotic process, cytolysis, pyroptosis, cell surface receptor signaling pathway and positive regulation of cell proliferation. The top 5 enriched cellular components were extracellular region, integral component of membrane, integral component of plasma membrane, external side of plasma membrane and plasma membrane. The top 5 enriched molecular function terms were receptor binding, identical protein binding, cytokine activity, tumor necrosis factor receptor binding and protein binding. The top 5 enriched KEGG pathways of the 29 DEPANRGs were necroptosis, allograft rejection, Ystemic lupus erythematosus, neutrophil extracellular trap formation, lipid and atherosclerosis.

### 3.3. PPI network establishment and correlation analysis of the DEPANRGs

**Figure 3.**
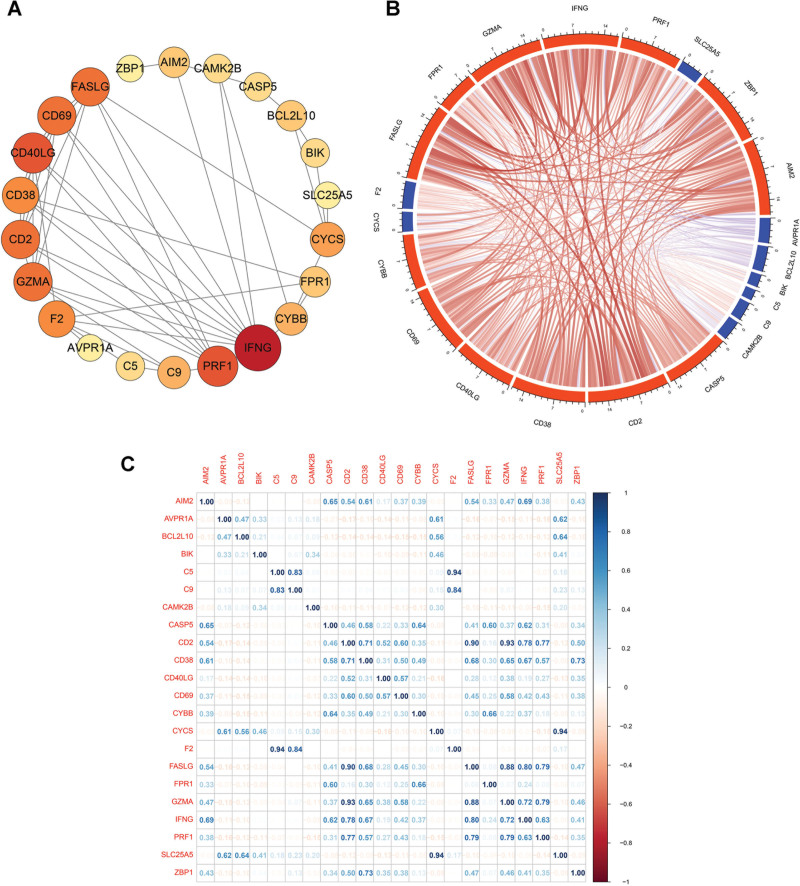
Interactions of the DEPANRGs. (A) The PPI network showing interactions among the DEPANRGs. (B) The chord diagram representing the correlation of the DEPANRGs. (C) Spearman analysis among the DEPANRGs. DEPANRG = differentially expressed PANoptosis-related gene, PPI = protein–protein interaction.

To further analyses the DEPANRGs, we downloaded the screened DEPANRGs of PPI network data from the STRING database (version 11.5), except 7 genes because of low confidence. PPI network was created and visualized by the Cytoscape software with results showing in Figure 3A. The size and color of the nodes represent the interaction degree. The chord diagram and Spearman analysis were adopted to investigate and visualized the relevance of the DEPANRGs (Fig. 3B and C).

### 3.4. Construction and verification of a DEPANRGs risk score

**Figure 4.**
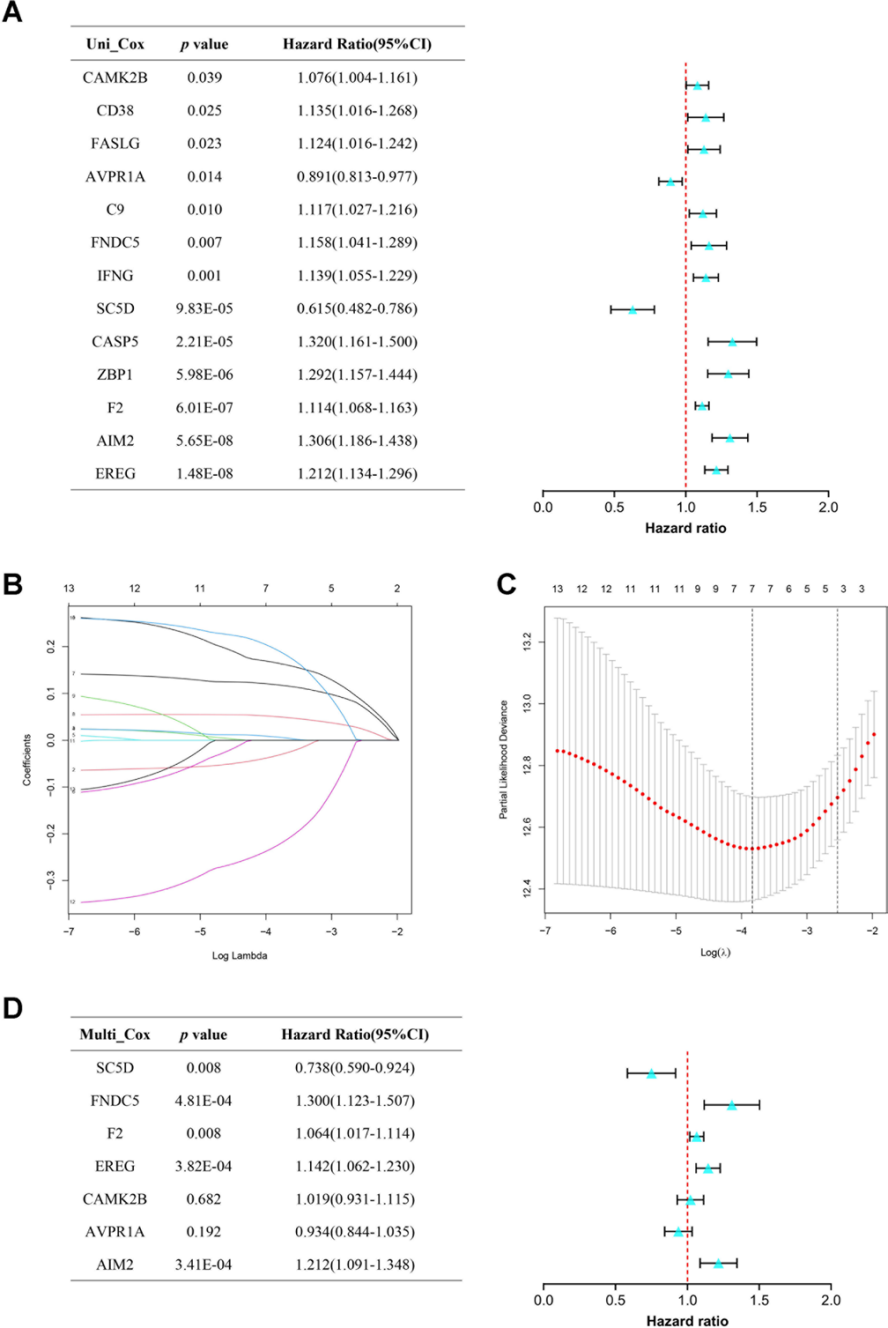
Identification of the optimal DEPANRGs to construct a risk score. (A) The forest plots showing 13 candidate DEPANRGs (*P* < .05) related to prognosis by univariate Cox analysis. (B) The penalty coefficients of the LASSO analysis. (C) The optimal *λ* value of the penalty parameter. (D) The forest plots showing 5 optimal DEPANRGs (*P* < .05) screened out for constructing a risk model by multivariate Cox analysis. DEPANRG = differentially expressed PANoptosis-related gene, LASSO = least absolute shrinkage and selection operator.


In order to identify the optimal prognostic genes for construction of a DEPANRGs risk score model, the univariate Cox analysis was initially applied and 13 DEPANRGs (C9, FNDC5, IFNG, CAMK2B, CD38, FASLG, AVPR1A, SC5D, CASP5, ZBP1, F2, AIM2, and EREG) were extracted by the inclusion criteria of *P* < .05 for following further study (Fig. 4A). Then, the LASSO analysis was employed on the 13 DEPANRGs in the training cohort, screening out 7 DEPANRGs based on the optimal *λ* value (Fig. 4B and C). Subsequently, we performed the multivariate Cox analysis on the 7 DEPANRGs, 5 of which were screened out for the construction of the DEPANRGs risk score model (including AIM2, EREG, F2, FNDC5, and SC5D) (Fig. 4D). Additionally, the coefficients of the 5 DEPANRGs were obtained in the process of performing multivariate Cox analysis. Finally, the 5 optimal DEPANRGs relevant coefficients and their expression levels were taken to calculate risk score according to the below equation.

$$\begin{array}{l} \begin{array}{llll} & Risk\;score=(0.20312)\times AIM2+(0.13146)\; & \times EREG+(0.06035)\times F2+(0.26019)\; & \times FNDC5+(-0.34442)\times SC5D.\; \end{array} \end{array}$$

### 3.5. Verification of the constructed risk model

**Figure 5.**
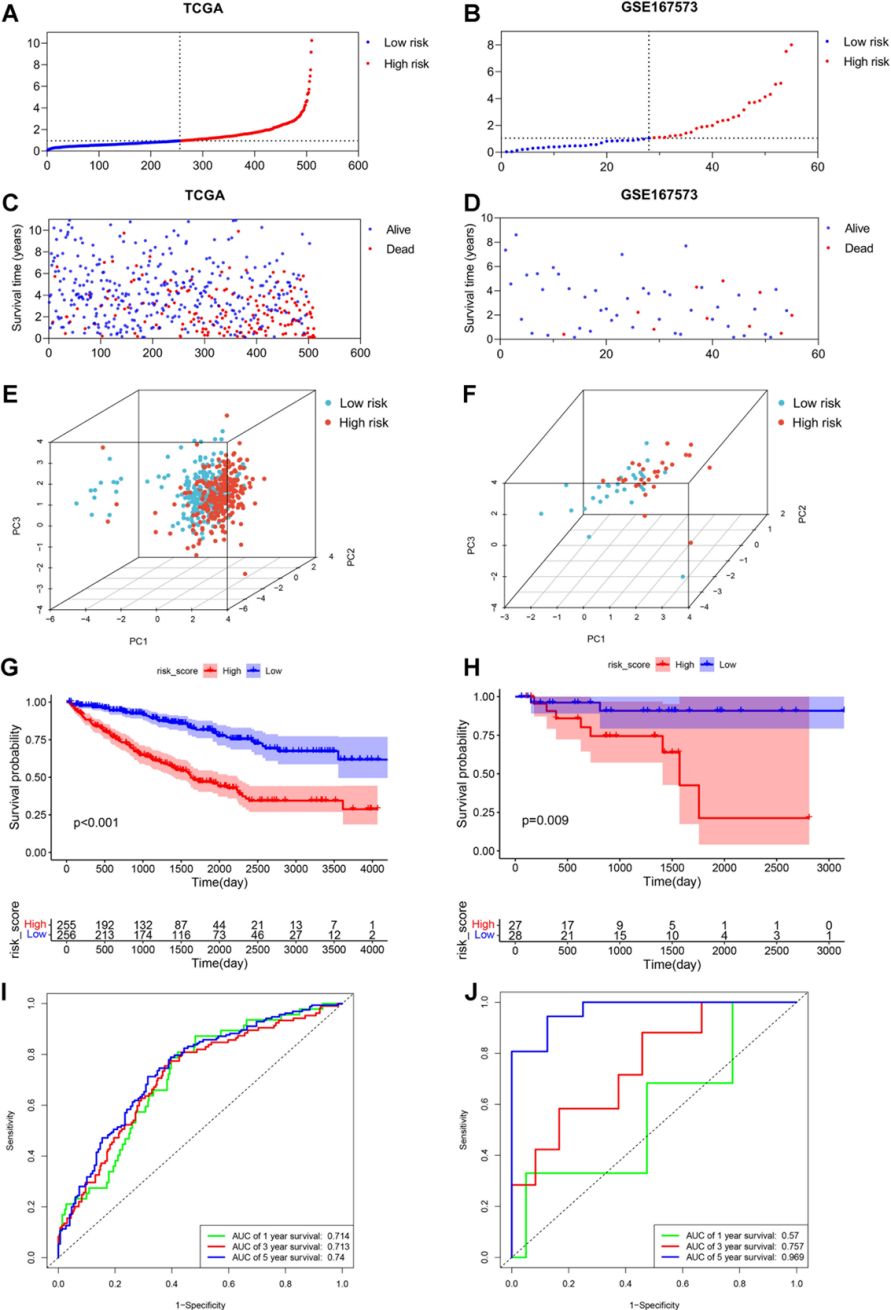
Prognosis analysis based on the risk score in the training (TCGA) and validation (GSE167573) cohorts. (A and B) Distribution of samples based on the risk score in the training (A) and validation (B) cohorts, respectively. (C and D) Survival status of samples in the training (C) and validation (D) cohorts, respectively. (E and F) Principal component analysis in the training (E) and validation (F) cohorts, respectively. (G and H) The K–M curves showing shorter overall survival in patients with high risk score in both the training (G) and validation (H) cohorts. (I and J) The ROC curves of 1-, 3-, and 5-year based on the risk score showing satisfied predictive value in the training (I) and validation (J) cohorts, respectively. K–M = Kaplan–Meier, ROC = receiver operating characteristic, TCGA = The Cancer Genome Atlas.

In order to determine the prediction capability of the risk model based on the 5 DEPANRGs, verification analysis was executed in the training and validation cohorts. The DEPANRGs risk scores for each patient were calculated based on the above equation. Taking the median risk score as the cutoff threshold, the ccRCC patients were categorized into low risk and high risk groups. The distribution of patients in low risk and high risk groups were represented in Figure 5A and B. The OS status of each ccRCC patient was exhibited by “*scatterplots*” package (Fig. 5C and D) in both cohorts, respectively. The 3D diagrams of principal component analysis showed that the 5 DEPANRGs signature performed powerful in discriminating samples (Fig. 5E and F). Kaplan–Meier survival curves were represented in Figure 5G and H indicating patients with low risk score presented a significantly more satisfactory prognosis in comparison to those with high risk score. To determine whether the risk score could predict prognosis well, ROC curves were implemented and displayed in Figure 5I and J. As presenting in the training cohort, the area under the ROC curves (AUC) were calculated for 1-, 3, - and 5-year OS prediction with results of 0.714, 0.713, and 0.740, respectively, demonstrating the risk score offered a satisfactory predictive efficiency of survival status in ccRCC patients. After conducted verification in the validation cohort, the corresponding AUC results were 0.570, 0.757, and 0.969, which also represented a reliable predictive capability for OS in ccRCC.

### 3.6. Verification of the risk score in clinical characteristic subgroups

**Figure 6.**
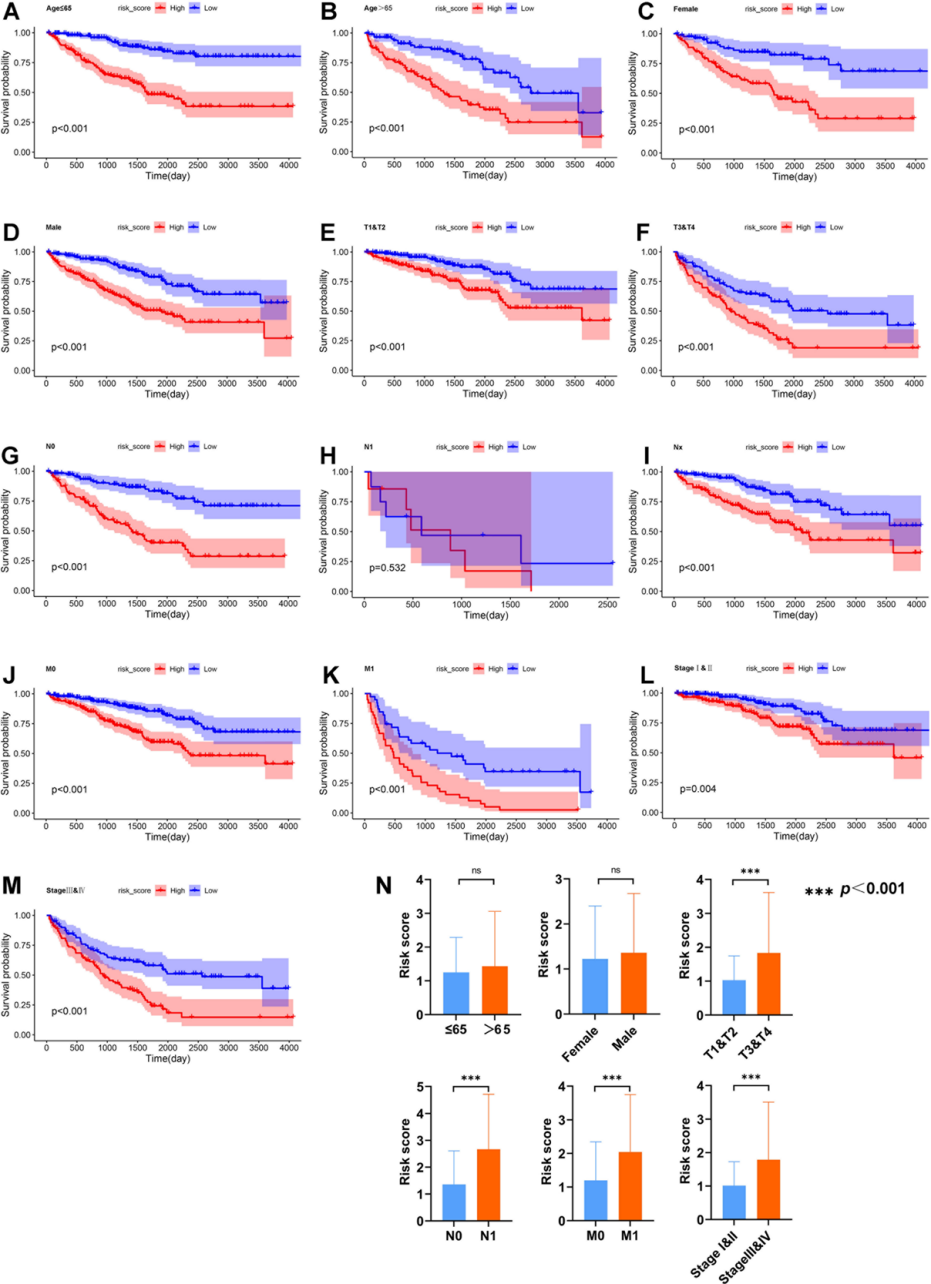
The K–M analysis based on the risk score in different clinical characteristic subgroups. (A) Age ≤ 65. (B) Age > 65. (C) Female. (D) Male. (E) T1 and T2. (F) T3 and T4. (G) N0. (H) N1. (I) Nx. (J) M0. (K) M1. (L) Clinical stage I and II. (M) Clinical stage III and IV. (N) Comparison of risk score in subgroups stratified by clinical characteristics. K–M = Kaplan–Meier.

For purpose of further evaluating the predictive effectiveness of the DEPANRGs risk model in clinical characteristic subgroups, K–M analysis was executed in subgroups stratified based on different clinical characteristics which contained age, gender, T, N, M, and clinical stage. We found the results of OS analysis were statistically distinct in almost clinical characteristic subgroups (*P* < .05) except the N1 stage subgroup (Fig. 6A–M). We also analyzed the distribution of the risk score in clinical characteristic subgroups, showing that the risk score was significantly different (*P* < .001) in several clinical characteristic subgroups (T, N, M, and clinical stage), but not subgroups of age and gender (Fig. 6N).

### 3.7. Correlation between the 5 DEPANRGs expression and the prognosis

**Figure 7.**
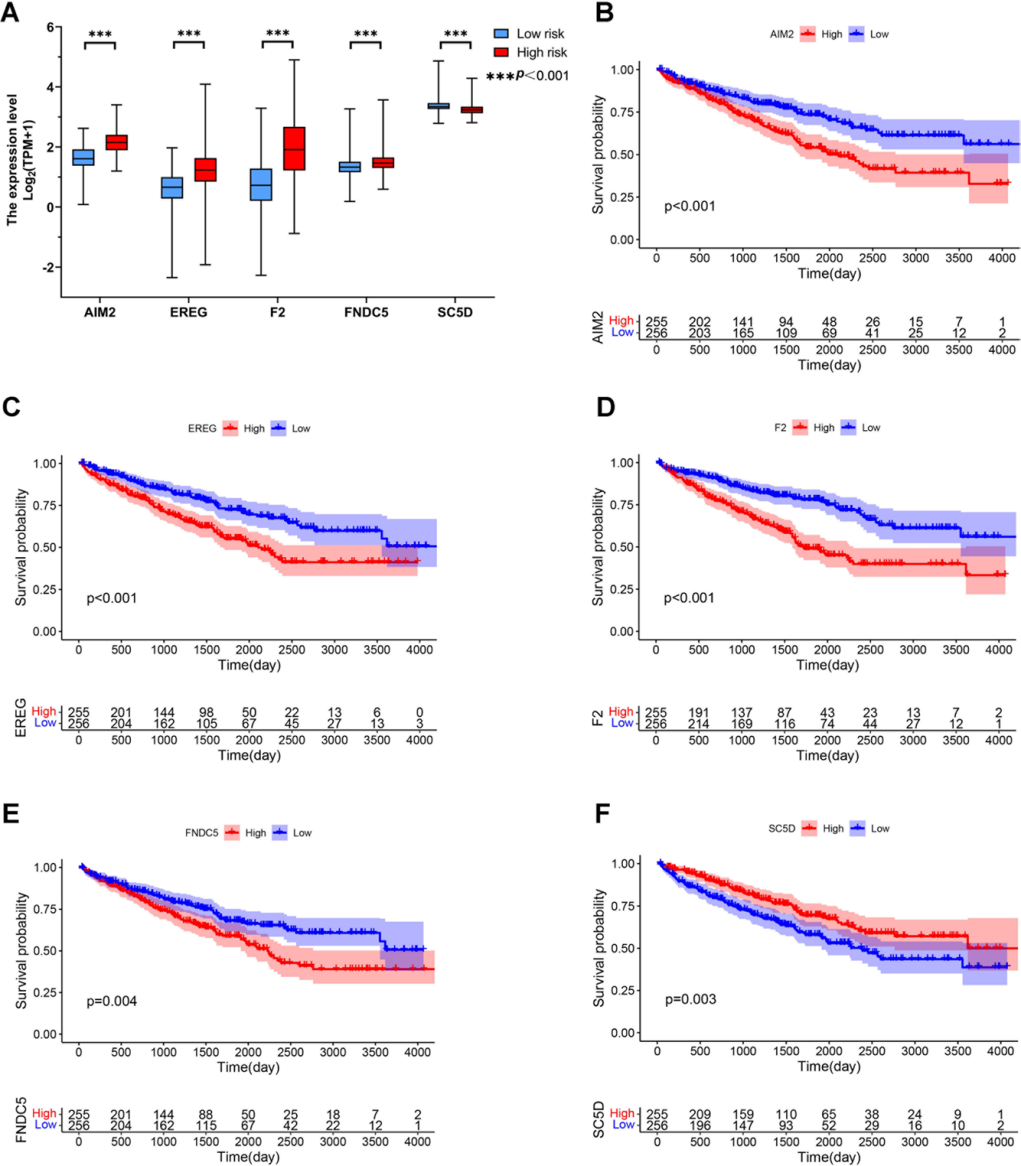
The correlation between the 5 DEPANRGs expression levels and OS. (A) Comparison of the 5 DEPANRGs expression levels between the high and low risk score groups. (B–F) The K–M analysis of AIM2 (B), EREG (C), F2 (D), FNDC5 (E) and SC5D (F), respectively. AIM2 = absent in melanoma 2, DEPANRG = differentially expressed PANoptosis-related gene, EREG = epiregulin, F2 = coagulation factor II, FNDC5 = fibronectin type III structural domain-containing protein 5, K–M = Kaplan–Meier, OS = overall survival, SC5D = sterol-C5-desaturase.

For further investigation, the expression of AIM2, EREG, F2, FNDC5, and SC5D were compared between patients in the low risk and high risk groups. The results are shown in Figure 7A, indicating that the expression levels of AIM2, EREG, F2, and FNDC5 were upregulated (*P* < .001) significantly, while SC5D was down-regulated (*P* < .001). The patients were stratified into subgroups according to the expression of the 5 DEPANRGs and K–M analysis was conducted in subgroups, respectively. We found upregulated expression of AIM2, EREG, F2, and FNDC5 was a significantly poor prognostic factor. Nevertheless, the upregulation of SC5D may contribute positively to the prognosis of ccRCC (Fig. 7B–F, *P* < .01).

### 3.8. Construction of a prognostic nomogram

**Figure 8.**
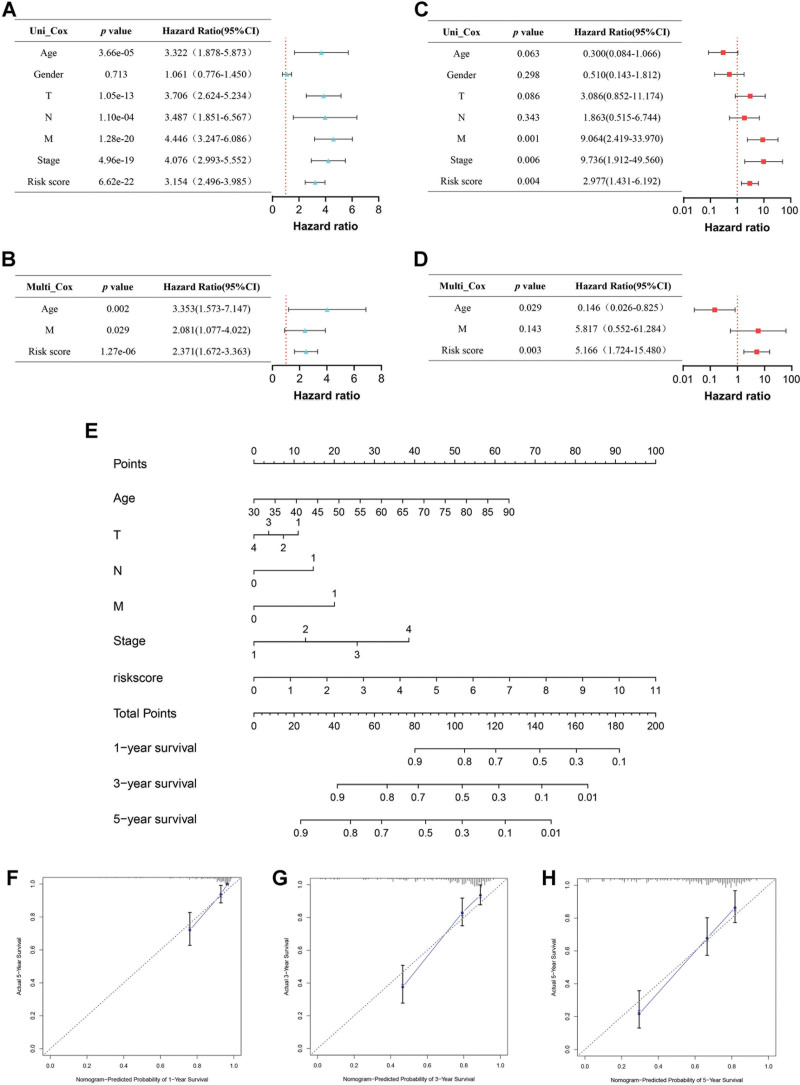
Construction of a nomogram for prognosis prediction. (A) Univariate Cox analysis between the risk score and clinical characteristics in the training cohort. (B) Multivariate Cox analysis in the training cohort. (C) Univariate Cox analysis between the risk score and clinical characteristics in the validation cohort. (D) Multivariate Cox analysis in validation cohort. (E) The constructed nomogram. (F–H) Calibration curves of 1-year (F), 3-year (G), 5-year (H) OS. OS = overall survival.

To identify the prognostic value of the risk score and clinical characteristics, the univariate and multivariate Cox analysis were performed in the training cohort from TCGA (Fig. 8A and B) and validation cohort (GSE167573) from GEO (Fig. 8C and D), respectively. We could learn from the outcomes that the risk score could be taken as an independent contributor for prognosis in both cohorts (the training cohort: univariate: hazard ratio (HR) = 3.154, *P* < .001; multivariate: HR = 2.371, *P* < .001; the validation cohort: univariate: HR = 2.977, *P* < .01; multivariate: HR = 5.166, *P* < .01). Additionally, we found that age (univariate: HR = 3.322, *P* < .001; multivariate: HR = 3.353, *P* < .01) and M stage (univariate: HR = 4.446, *P* < .001; multivariate: HR = 2.081, *P* < .05) were recognized as independent predictors for OS in the training cohort. Integrating risk score and above clinical characteristics (except gender), a nomogram were created and applied for further assessing survival rate of 1-, 3-, and 5-year for ccRCC patients (Fig. 8E). Calibration curves were represented in Figure 8F–H showing considerable accuracy in prediction.

### 3.9. Examination the DEPANRGs and immune infiltration

**Figure 9.**
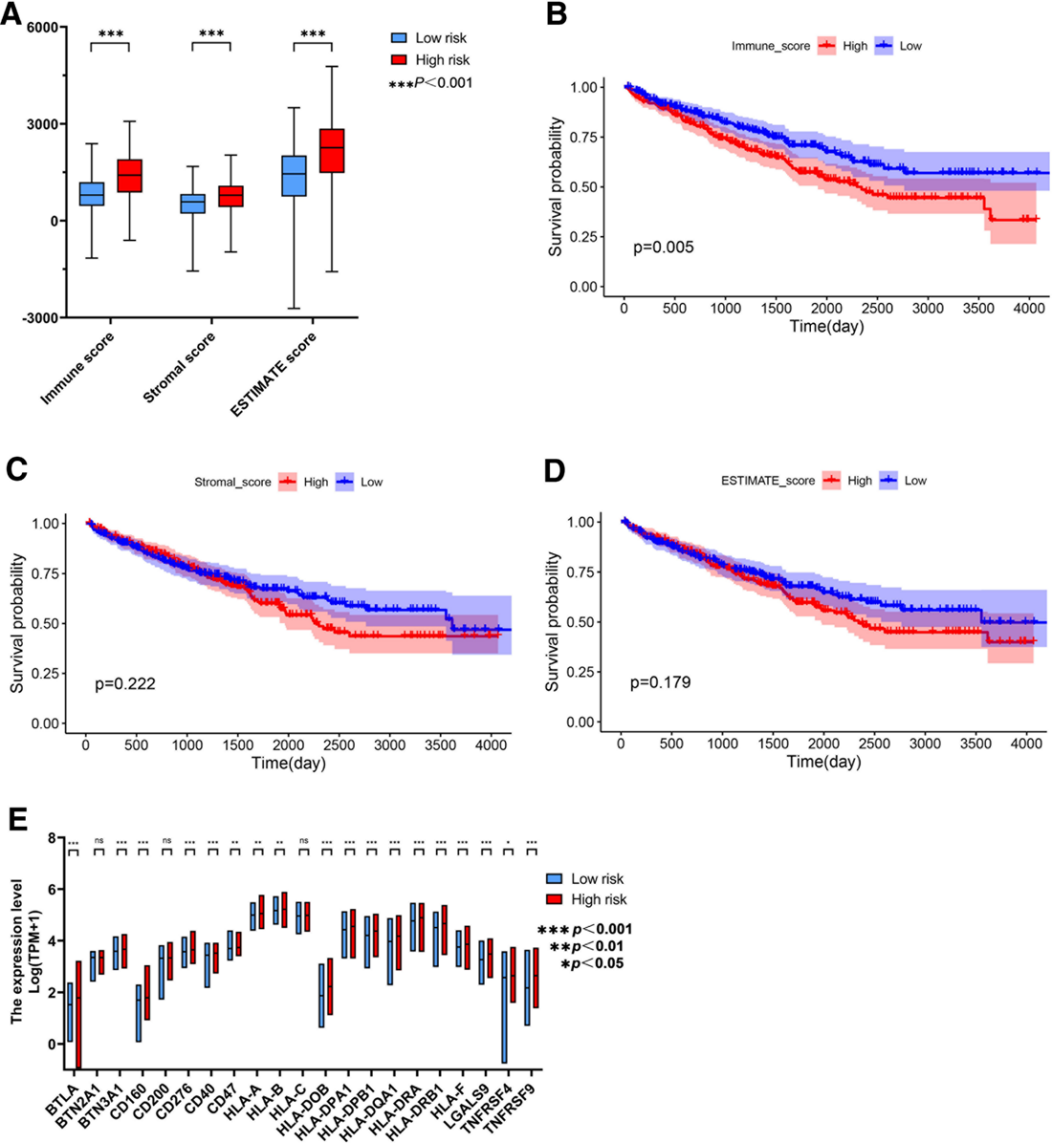
Immune correlation analysis. (A) Comparison of the ESTIMATE, stromal and immune scores between the low and high risk score groups (*P* < .001). (B) The K–M analysis based on immune score (*P* < .01). (C) The K–M analysis based on stromal score. (D) The K–M analysis based on ESTIMATE score. (E) Analysis of immune checkpoints expression. K–M = Kaplan–Meier.

**Figure 10.**
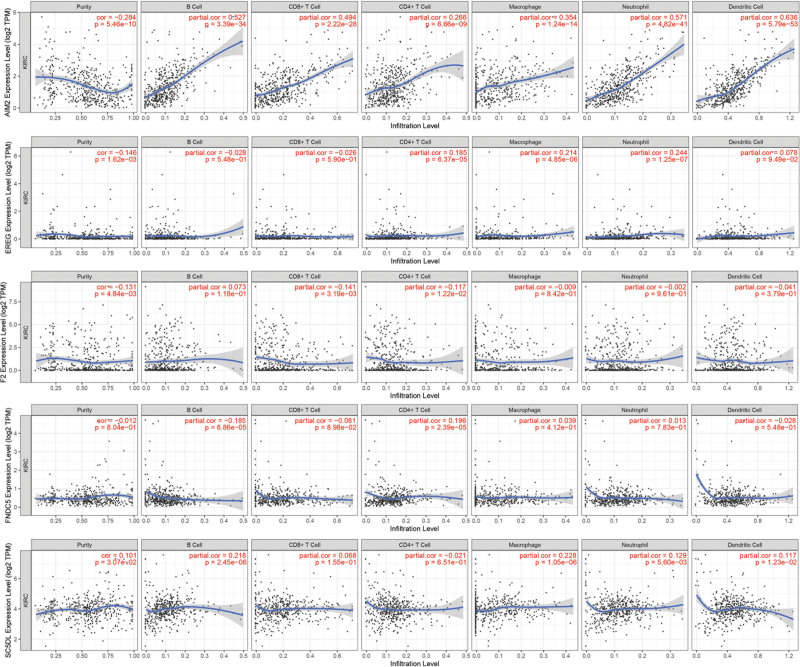
Correlation between the 5 prognostic DEPANRGs and 6 types of immune infiltration cells. DEPANRG = differentially expressed PANoptosis-related gene.

The ESTIMATE, stromal and immune scores corresponding to patients from the TCGA database were employed to analyze the correlation between tumor microenvironment (TME) components and OS. As is shown in Figure 9A, the ESTIMATE, stromal and immune scores were significantly high in the high risk group (*P* < .001). Kaplan–Meier analysis based on these scores were conducted (Fig. 9B–D), and our results revealed that high immune score represented a robust relationship with the worse prognosis in ccRCC (*P* < .05). Additionally, according to our findings, there was a higher activation of most immune checkpoints like HLA-DOB and TNFRSF9 among individuals with high risk (Fig. 9E). Moreover, we assessed the correlation between the 5 prognostic DEPANRGs and 6 types of immune infiltration cells (including B cell, CD8+T cell, CD4+T cell, macrophage, neutrophil, and dendritic cell) through the TIMER database comprehensively with results represented in Figure 10.

## 4. Discussion

ccRCC, as a common malignant tumor, has received more attention, and considerable advances have been made in studies on the mechanisms of its pathogenesis and progression.^[25–28]^ On the basis of previous multi-omics studies, including genomics, lipidomics, epigenetics, metabolomics, and proteomics, ccRCC has been identified as a metabolic-related tumor involving aberrant alterations in multiple cellular metabolic pathways and biological processes.^[29,30]^ For instance, with adequate oxygen provision, tumor cells principally generate energy through glycolysis, which is very different from the mitochondrial oxidative phosphate pathway of energy acquisition in normal cells.^[31]^ In addition, other studies have implicated that both the disruption of mitochondrial metabolism and redox homeostasis are involved in the invasion of ccRCC cells.^[32,33]^ Furthermore, overexpression of lipid droplet-related proteins could cause aberrant lipid enrichment and storage in tumor cells, which is a characteristic alteration in ccRCC.^[34]^ The above studies on metabolic profiles indicated that abnormal alterations of metabolism may be tightly involved in the progression and poor prognosis of ccRCC.

The first proposal of PANoptosis by Malireddi et al^[35]^ in 2019 has provided a completely new approach to the understanding of cell death, which combined the core molecular features of pyroptosis, apoptosis and necroptosis. Numerous studies on PANoptosis have been conducted and provided considerable evidence suggesting that PANoptosis may contribute significantly to the host fighting against various pathogens including viruses, bacteria, fungi, and even parasites.^[36]^ Additionally, considering researches of PANoptosis on TME alteration and tumorigenesis, PANoptosis has provided more potential strategies for tumor treatment. For example, Karki study^[37]^ showed that knockdown of interferon regulatory factor 1 could significantly inhibit the activation of GSDMD (pyroptosis), CASP3/7 (apoptosis) and MLKL (necroptosis), thereby promoting cell death in colitis-associated CRC. This indicated that interferon regulatory factor 1, as an important upstream regulatory molecular of PANoptosis, could restrict the tumorigenesis by mediating the activation of PANoptosis. Another investigation on PANoptosis demonstrated that adenosine deaminase acting on RNA 1 could suppress the occurrence of PANoptosis, which could in turn promote the tumorigenesis and progression of melanoma and CRC.^[38]^ Some other researches on the role of PANoptosis in tumorigenesis have also confirmed its involvement in a wide range of tumors, including breast carcinoma, liver carcinoma, nasopharyngeal carcinoma and so on.^[39–41]^

A growing number of studies have revealed a significant correlation and crosstalk between PANoptosis and metabolism.^[29,30,42–44]^ For instance, abnormality of intracellular lipid metabolism can cause alterations of endoplasmic reticulum homeostasis, which in turn promotes tumor progression by suppressing apoptosis.^[30,33,43]^ Additionally, mitochondrial oxidative stress could promote cellular pyroptosis by promoting CASP1-mediated cleavage of GSDMD.^[43]^ In turn, activated GSDMD could further exacerbate mitochondrial dysfunction by forming pores in the mitochondrial membrane. Inactivated BAK is normally located in the mitochondria. When BAK is activated, BAX located in cytoplasm will aggregate in mitochondria.^[43]^ BAX/BAK then promotes the release of Cyt C, causing a cascade reaction in the apoptotic pathway. Moreover, upregulation of phosphoglycerate mutase family member 5, mediated by phosphorylation of RIPK3 and MLKL, could suppress glutathione synthesis and cause mitochondrial dysfunction.^[33,43]^ According to the above studies, PANoptosis could alter cellular metabolic status by regulating mitochondrial function, and this suggests that PANoptosis may act an important role in regulating cell death pathways via modulation of cellular metabolism and biological processes.

All of the above mentioned investigations provided us with new perspectives on the understanding of PANoptosis’s role in tumor occurrence and development, which attracted our interest. Nevertheless, current researches have not elucidated the underlying mechanism on PANoptosis occurring in ccRCC. Therefore, we implemented this study to construct a DEPANRGs signature for predicting the prognosis, which may contribute to the new findings on potential strategies for the therapy of ccRCC patients.

After analyzing the transcriptome data from the TCGA database in current study, we screened out 29 DEPANRGs, which were candidate genes contributing to OS of ccRCC patients. After functional analysis of these candidate genes in DAVID database, we found a significant and positive involvement of these DEPANRGs in apoptosis, pyroptosis and necroptosis processes. For further study, 13 out of the 29 DEPANRGs were identified as the potential hub genes correlated to OS by using the univariate Cox analysis. Then, we performed further analysis to select key prognosis-related genes by the LASSO and multivariate Cox analysis sequentially, resulting in 5 hub genes (AIM2, EREG, F2, FNDC5, and SC5D) being confirmed to construct a risk model. The results of K–M analysis discovered that patients with high risk score had significantly poor prognosis in both the training and validation cohorts. This indicated that the risk score offered reliable predictive power for OS in ccRCC. ROC curves were evaluated by integrating OS and risk level. The outcomes of AUC for 1-, 3-, and 5-year respectively were 0.714, 0.713, and 0.740 in the training cohort, which implied that the risk score held a highly plausible prognostic capability. These predictive results were verified in the validation cohort, but the AUC of 1-year OS was 0.570 which was below 0.7 implying this was not ideal.^[45]^ This may result from the limited sample size of the validation cohort, so a validation cohort with a large sample size may be more effective. Based on an integrated analysis combining risk score and clinical characteristics, the risk score was conclusively discriminated to be an independent prognostic indicator in the training cohort, while the consistent outcomes in the validation cohort were simultaneously verified. Furthermore, the nomogram and calibration curves calculated based on the risk score and clinical characteristics also represented powerful predictive value and diagnostic significance. All the above results suggested that the constructed PANoptosis-related gene signature can accurately and effectively predict prognosis.

Previous studies have confirmed that the TME is of great heterogeneity and complexity, and its alteration plays a vital role in enhancing both the malignant tumor cell growth and invasiveness.^[46]^ The high complexity of the TME lies in the presence of numerous different types of cellular constituents, such as tumor cells, fibroblasts, immune cells, and endothelial cells.^[47–51]^ The presence of complex crosstalk and interactions within these cells could lead to increasing angiogenesis, metabolic reprogramming, as well as promotion of tumor cell growth, invasion, and metastasis in ccRCC tumor tissues.^[52]^ Moreover, in previous studies, activation of the mammalian target of rapamycin pathway could promote angiogenesis by promoting glycolysis and lactate metabolism.^[47,52]^ Concurrently, this pathway could enhance the degree of immune cell infiltration by modulating the production of cytokines and chemokines.^[53–56]^ These evidences demonstrated that certain specific metabolic pathways could modulate angiogenesis as well as immune infiltration in tumor tissues.

In view of the above studies, we conducted correlation analysis between these 5 DEPANRGs (AIM2, EREG, F2, FNDC5, and SC5D) expression and OS, TME, immune infiltration. The outcomes showed that the expression of the 5 DEPANRGs demonstrated a close correlation with OS and immune infiltration, implying patients of ccRCC may have a good response to immunotherapy and benefit from it. This may provide novel treatment strategies and insight for ccRCC patients. This view was consistent with some previous studies.^[57]^ Additionally, in the present study, there was significant variability in the comparison of ESTIMATE scores and immune checkpoints expression between the 2 risk subgroups constructed by the 5DEPANRGs. Moreover, our study also found that 5DEPANRGs have a high degree of correlation with diverse immune cells. This implied that PANoptosis may promote angiogenesis, alter the TME and enhance the level of immune infiltration in tumor tissues by modulating cellular metabolic pathways.

Of these 5 DEPANRGs, AIM2 (absent in melanoma 2), as the most typically characterized member of the AIM2-like receptors, mediates the recruitment of apoptosis-associated specklike protein containing a CARD and the assembly of AIM2 inflammasome.^[58,59]^ AIM2 can also activate CASP1 and result in the release of effector cytokines, driving cell death.^[59]^ Moreover, previous studies have showed that AIM2 promotes tumorigenesis in cutaneous squamous cell carcinoma,^[60]^ but exerts inhibition effect on liver cancer.^[61]^ In present study, upregulation of AIM2 predicted worse prognosis of ccRCC patients, that was consistent with previous studies.^[62,63]^

Epiregulin (EREG) has been proved to contribute to the tumor initiation, progression and metastasis in a variety of tumors through activating EREG/EGFR pathway.^[64]^ For instance, the occurrence of EREG upregulation has been confirmed in non-small-cell lung cancers, which strongly resulted in shorter OS.^[65]^ Another study revealed that EREG may act as a risk prognostic factor and a predictive marker in CRC, showing a better prognostic outcome in patients accepting neoadjuvant chemoradiotherapy.^[66]^ Moreover, the binding of EREG to EGFR has been identified to be involved in carcinogenic function of tumors, with examples of cervical cancer^[67]^ and gastric cancer.^[68]^ The present study showed that the upregulation of EREG may act a detrimental role in ccRCC.

Coagulation factor II (F2), encoding thrombin, is located on chromosome 11p11 and is correlated with susceptibility to systemic lupus erythematosus.^[69]^ In our study, the upregulation of F2 may contribute negative impact to rehabilitation of ccRCC. Unfortunately, there are few studies on F2 in tumors, suggesting further studies and more evidence on the role of F2 in ccRCC are required.

Fibronectin type III structural domain-containing protein 5 (FNDC5), which is regarded as a prohormone, is cleaved proteolytically followed by the release of irisin.^[70]^ Liu study^[71]^ revealed that the development of hepatocellular carcinoma may be promoted and accelerated by the upregulated expression of FNDC5, probably though suppressing the activation of NF-κB pathway and NLRP3. Similar to the above finding, upregulation of FNDC5 expression may cause a significantly negative prognostic effect, which was demonstrated in our study. Nevertheless, FNDC5/irisin has been shown to have suppressive effect on invasion of lung cancer cells in vitro studies.^[72]^ Additionally, Altay study^[73]^ also suggested that FNDC5 may act as a more sensitive and accurate marker in the diagnosis of renal cancer.

Sterol-C5-desaturase (SC5D) was found to be significantly positive with the progression of gastric cancer.^[74]^ However, our results revealed that the upregulation of SC5D contributed to better prognosis implying SC5D may be a protective indicator in ccRCC. Therefore, it is essential to conduct more studies to reveal the potentially specific mechanism of SC5D and clarify whether it could be used as a protective factor in tumors.

In our study of the PANoptosis-related genes in ccRCC, we obtained many satisfied results and advances. However, there are several limitations that need to be noticed and acknowledged in our presented research. Firstly, the gene expression data we used for analysis was obtained from only 2 databases. Our findings are needed to be validated in other independent alternative databases. Secondly, we implemented a preliminary investigation on PANoptosis, which was just a bioinformatic study. Our study did not include independent experimental validation such as the employment of cell lines, animal models and clinical tissue samples, which would likely weaken the reliability of this study. Therefore, our study requires further in vitro and in vivo experiments to validate and support our conclusions by transcriptomics, proteomics, lipidomics, and so on.

## 5. Conclusions

In conclusion, we identified 5 PANoptosis-related genes that were strongly associated with the prognosis of ccRCC patients by a comprehensive bioinformatics analysis. Based on these 5 genes (AIM2, EREG, F2, FNDC5, and SC5D), a risk score and a nomogram were constructed to predict the prognosis of ccRCC patients with robust reliability. However, further researches are needed to reveal the underlying mechanism of these PANoptosis-related genes in regulating the development of ccRCC.

## Acknowledgments

All authors greatly acknowledgments the TCGA, GEO, ESTIMATE, TIMER, and STRING databases for free downloaded data.

## Author contributions

**Conceptualization:** Dezhi Yue, Xiaoqiang Liu.

**Data curation:** Dezhi Yue, Congzhe Ren, Hu Li.

**Formal analysis:** Dezhi Yue, Congzhe Ren.

**Methodology:** Hu Li.

**Supervision:** Xiaoqiang Liu.

**Validation:** Dezhi Yue, Congzhe Ren, Xiaoqiang Liu.

**Visualization:** Hu Li.

**Writing – original draft:** Dezhi Yue.

**Writing – review & editing:** Xiaoqiang Liu.
